# Galectin-14 Promotes Trophoblast Migration and Invasion by Upregulating the Expression of MMP-9 and N-Cadherin

**DOI:** 10.3389/fcell.2021.645658

**Published:** 2021-03-16

**Authors:** Miaomiao Wang, Yuqing Xu, Peng Wang, Yanfei Xu, Pengzhen Jin, Zaigui Wu, Yeqing Qian, Long Bai, Minyue Dong

**Affiliations:** ^1^Women’s Hospital, School of Medicine, Zhejiang University, Hangzhou, China; ^2^Key Laboratory of Women’s Reproductive Health of Zhejiang Province, Hangzhou, China; ^3^Key Laboratory of Reproductive Genetics, Ministry of Education, Zhejiang University, Hangzhou, China

**Keywords:** early pregnancy loss, preeclampsia, epithelial-mesenchymal transition (EMT), matrix metalloproteinases (MMPs), Akt

## Abstract

Galectin-14 is specifically expressed in placental trophoblasts, and its expression is reduced in trophoblasts retrieved from the cervix of women destined to develop early pregnancy loss. However, the roles of galectin-14 in regulating trophoblasts and in the pathogenesis of pregnancy complication have never been investigated. In the current research, we aimed to investigate the roles of galectin-14 in the regulation of trophoblasts. Tissues of the placenta and villi were collected. Primary trophoblasts and human trophoblast cell line HTR-8/SVneo were used. Western blotting and RT-PCR were used to quantify gene expression. The siRNA-mediated galectin-14 knockdown and lentivirus-mediated overexpression were performed to manipulate the gene expression in trophoblasts. Transwell migration and invasion assays were used to evaluate cell migration and invasion capacity. Gelatin zymography was used to determine the gelatinase activity. Galectin-14 was significantly decreased in the villi of early pregnancy loss and the placenta of preeclampsia. Knockdown of galectin-14 in primary trophoblasts inhibited cell migration and invasion, downregulated the expression of matrix metalloproteinase (MMP)-9 and N-cadherin, the activity of MMP-9, and decreased the phosphorylation of Akt. Meanwhile, the overexpression of galectin-14 in HTR-8/SVneo promoted cell migration and invasion, upregulated the expression of MMP-9 and N-cadherin, the activity of MMP-9, and increased the phosphorylation of Akt. Increased Akt phosphorylation promoted cell migration and invasion and upregulated the expression and activity of MMP-9, while decreased Akt phosphorylation inhibited cell migration and invasion and downregulated the expression and activity of MMP-9. Thus, galectin-14 promotes trophoblast migration and invasion by enhancing the expression of MMP-9 and N-cadherin through Akt phosphorylation. The dysregulation of galectin-14 is involved in the pathogenesis of early pregnancy loss and preeclampsia.

## Introduction

Placenta is a temporary organ connecting the fetus and her mother. The trophoblast is the main cellular component of the placenta. The normally functioning trophoblast is crucial for the establishment and maintenance of pregnancy, development of the placenta, remodeling of the spiral artery, and subsequently fetal growth and maturation (Turco and Moffett, 2019). Dysregulation of trophoblasts is associated with pregnancy complications including early pregnancy loss (EPL) (Huppertz, 2020) and preeclampsia (Huppertz, 2018).

The function of trophoblasts is regulated by a group of genes, especially placenta-specific genes including PLAC1 and syncytin (Rawn and Cross, 2008). These genes regulate trophoblastic migration, invasion, proliferation, apoptosis, and metabolism (Bolze et al., 2017; Wan et al., 2019). Galectins are among these genes.

Galectins are a family of soluble β-galactoside-binding proteins and consist of 20 members (Blois et al., 2019). Recently, several galectin family members including galectin-1, galectin-3, galectin-9, and galectin-13 were found to be dysregulated in pregnancy complications. The placental expressions of galectin-1 and galectin-13 were decreased in early pregnancy loss and preeclampsia (Liu et al., 2006; Freitag et al., 2013; Than et al., 2014), whereas the expressions of galectin-3 and galectin-9 were increased in preeclampsia (Jeschke et al., 2007; Blois et al., 2019).

Galectin-14, a recently identified galectin family member, is specifically expressed in placenta with over 12,000 times expression level compared with other tissues (Than et al., 2009). Using immunocytochemistry, a decrease in galectin-14 was observed in trophoblasts retrieved from the cervix at the gestational age of 5–10 weeks before the onset of early pregnancy loss (Fritz et al., 2015). However, the regulatory roles of galectin-14 in trophoblasts have never been investigated yet.

In the current study, the placental expressions of galectin-14 in early pregnancy loss and severe preeclampsia were determined, the effects of galectin-14 on the migration and invasion of trophoblast were observed, and mechanisms behind were explored.

## Materials and Methods

### Subjects

Villi were collected from 28 women who were undergoing artificial abortion and 28 cases of early pregnancy loss. Placenta tissues were collected from 20 women of normal pregnancy and 20 with severe preeclampsia at Cesarean sections.

Early pregnancy loss was diagnosed if the ultrasound examination showed an empty sac with a mean diameter over 25 mm or an embryo with a crown–rump length over 7 mm without heartbeat within the first 12 6/7 weeks of gestation (American College of Obstetricians and Gynecologists’ Committee on Practice Bulletins—Gynecology, 2018). Women undergoing artificial abortions with a similar gestational age served as control. The pregnancies complicated by autoimmune disease, genital malformation, history of exposure to poison or X-ray, and other acute or chronic diseases were excluded. Severe preeclampsia was diagnosed according to the criteria recommended by the American College of Obstetricians and Gynecologists (ACOG) (American College of Obstetricians and Gynecologists, 2019). Exclusion criteria were multiple pregnancy, pregnancy complicated by chronic hypertension, diabetes mellitus, and other acute or chronic diseases. Demographic characteristics of the cases were described in Tables 1, 2.

**TABLE 1:** Demographic characteristics of abortion and early pregnancy loss.

	Normal pregnancy	Early pregnancy loss	*p*-value	
*N*	28	28		
Maternal age (years; mean ± SD)	29.93 ± 4.944	31.07 ± 4.430	0.366	>0.05
Gestational age at surgery (days; mean ± SD)	58.29 ± 7.378	60.50 ± 7.758	0.279	>0.05

**TABLE 2:** Demographic characteristics of normal terminal pregnancy and preeclampsia.

	Normal pregnancy	Preeclampsia	*p*-value	
*N*	20	20		
Maternal age (years; mean ± SD)	34.4 ± 4.3	31.5 ± 5.4	0.063	>0.05
BMI (kg/m^2^; mean ± SD)	26.81 ± 3.94	29.45 ± 4.15	0.046	*
Gestational age at delivery (weeks; mean ± SD)	38.0 ± 1.4	35.0 ± 3.1	0.0001	***
Blood pressure				
Systolic (mmHg; mean ± SD)	117.9 ± 11.9	167.2 ± 12.3	0.0001	***
Diastolic (mmHg; mean ± SD)	74.8 ± 7.5	103.4 ± 10.2	0.0001	***
Proteinuria	−	−(2 cases)		
		+∼++(9 cases)		
		+++∼++++(9 cases)		
Birth weight (g; mean ± SD)	3,305.0 ± 497.0	2,398.3 ± 751.0	0.0001	***
Fetal gender	Female: 12	Female: 11		>0.05
	Male: 8	Male: 9		

*p < 0.05; ***p < 0.001.

This study was approved by the Institutional Ethical Committee of Women’s Hospital, School of Medicine, Zhejiang University, and informed consents were obtained.

### Cell Culture

HTR-8/SVneo, Swan-71, JAR, JEG-3, and BeWo were cultured in RPMI 1640 medium, DMEM/F12 medium, or Ham’s F-12K medium (Thermo Fisher Scientific, Waltham, United States) with 10% fetal bovine serum (FBS) and antibiotics (100 U/ml penicillin and 100 mg/ml streptomycin) at 37°C with 5% CO_2_. HTR-8/SVneo was obtained from ATCC (Rockville, MD, United States) and authenticated by STR profiling.

Primary extravillous trophoblasts (EVTs) were isolated from the villi of artificial abortion. Briefly, tissues were washed with cold phosphate buffered saline (PBS). Amniotic membrane and decidua were removed, and villi were minced. The fragments were resuspended with high-glucose DMEM with 10% FBS. After the cells attached on the bottom, bottles were washed with PBS gently, and fresh medium was added. EVT purity was examined by immunofluorescence staining of HLA-G and CK-7. Cells with purity over 90% and within four passages were used in the experiments.

### Gene Knockdown and Overexpression

Cells were transfected with a SMARTpool siRNA or scramble siRNA (GenePharma, Shanghai, China) using Lipofectamine RNAiMAX transfection reagent (Thermo Fisher Scientific, Waltham, United States) according to the manufacturer’s instructions.

For overexpression, cells were transfected with lentivirus carrying overexpression vector or empty vehicle according to the proper multiplicity of infection (MOI). Here, 5–8 mg/ml polybrene (Cell-Land, Hangzhou, China) was added in the culture medium.

### Cellular Immunofluorescence Assay

Adherent cells were washed with PBS three times and fixed with 4% paraformaldehyde (Biosharp, Hefei, China) for 15 min. Then, cells were permeabilized with 0.1% Triton X-100 (Sigma-Aldrich, Temecula, United States) for 10 min, blocked for 30 min in 5% bovine serum albumin (BSA) at room temperature, and incubated with primary antibodies against CK7 (1:100), HLA-G (1:100), and galectin-14/LGALS14 (1:100) at 4°C overnight. After rinsing with PBS, cells were incubated with a secondary fluorescein isothiocyanate (FITC) antibody (1:400 MultiSciences Biotech Co., Hangzhou, China) for 1 h at room temperature. Mounting medium with 4’,6-diamidino-2-phenylindole (DAPI) was added in the amount of 1 drop per hole and rinsed off in 5 min. The fluorescence signal was detected by fluorescence microscope (Leica, Wetzlar, Germany).

### Western Blotting

Tissues were homogenized in 200 μl radioimmunoprecipitation assay (RIPA) lysis buffer containing protease inhibitor cocktail (Selleck, Houston, United States) on ice. Cells were lysed with 50 μl RIPA lysis buffer containing protease inhibitor cocktail on ice.

Lysates were centrifuged at 15,000 *g* at 4°C for 30 min. The protein concentration was quantified using BSA kit (MultiSciences Biotech Co., Hangzhou, China). In total, 30 μg protein was separated by 12% sodium dodecyl sulfate (SDS)-polyacrylamide gel and transferred onto a polyvinylidene fluoride (PVDF) membrane (Bio-Rad, Hercules, United States) with ice. Membranes were blocked for 1 h with Tris-buffered saline containing 0.1% Tween 20 (TBS/T) containing 5% BSA (Solarbio, Beijing, China) at room temperature. Then, membranes were incubated with primary antibodies against galectin-14/LGALS14 (1:1,000), VE-cadherin (1:1,000), N-cadherin (1:1,000), matrix metalloproteinase 9 (MMP-9; 1:1,000), tissue inhibitor of matrix metalloproteinase 1 (TIMP-1; 1:1,000), MMP-2 (1:1,000), TIMP-2 (1:1,000), phospho-Akt (1:1,000), Akt (1:1,000), and glyceraldehyde 3-phosphate dehydrogenase (GAPDH; 1:1,000) at 4°C overnight. After washing in TBS/T, membranes were incubated with horseradish peroxidase (HRP)-labeled secondary antibodies (1:5,000) for 1 h. The bands were visualized with enhanced chemiluminescence (ECL) kit (Fdbio Science, Hangzhou, China) by autoradiography (ImageQuant TL Software, GE Healthcare, Pittsburgh, United States).

### Reagents and Antibodies

The phosphatidylinositol 3-kinase (PI3K) inhibitor LY294002 and Akt agonist SC79 were purchased from Selleck (Houston, United States). Rabbit monoclonal anti-LGALS14 (ab150427), rabbit monoclonal anti-VE-cadherin (ab33168), rabbit monoclonal anti-N-cadherin (ab76011), mouse monoclonal anti-MMP-9 (ab119906), rabbit monoclonal anti-TIMP1 (ab109125), rabbit monoclonal anti-MMP-2 (ab92536), rabbit monoclonal anti-TIMP-2 (ab180630), rabbit monoclonal anti-GAPDH (ab181602), rabbit monoclonal anti-cytokeratin 7 (ab181598), and mouse monoclonal anti-HLA G (ab52455) antibodies were purchased from Abcam (Cambridge, United Kingdom). Rabbit monoclonal anti-p-Akt (Ser473) (9271) and rabbit monoclonal anti-Akt (9272) antibodies were purchased from Cell Signaling Technology (Danvers, United States). Goat anti-mouse IgG (H + L) HRP and goat anti-rabbit IgG (H + L) HRP secondary antibodies were purchased from MultiSciences Biotech Co. (Hangzhou, China).

### RT-PCR and RT-qPCR

RNA isolation and reverse transcription were performed as previously described (Yan et al., 2016).

Reverse transcription semiquantitative PCR (RT-PCR) was performed using a PTC 200 thermal cycler, and each 25-μl reaction contained 1 × GoldStarTaq Master Mix, 100 ng cDNA, and 400 nmol/L (nM) of each specific primer. Amplifications were performed as follows: 10 min at 95°C, 30 amplification cycles (30 s at 95°C, 30 s at 55°C, and 1 min at 72°C) and final extension for 5 min at 72°C. PCR products were analyzed by 2% ethidium bromide staining agarose gel electrophoresis, quantified by densitometry (Image Lab software, Bio-Rad Laboratories Inc., Hercules, United States) and normalized against GAPDH.

The quantitative PCR (qPCR) was performed in ViiA 7 Real-Time PCR System (Thermo Fisher Scientific, Waltham, United States) by using TB Green Premix Ex Taq (Tli RNase H Plus) Kit (Takara Bio Inc., Shiga, Japan) according to the manufacturer’s instructions.

The sequences of primers were as follows:

Galectin-14 forward 5′-CATCACTACCCGTACCATACA CACT-3′,
reverse 5′-GCTGTGGGTCCTTGACAAAAGTGAG-3′;
GAPDH forward 5′-GGCAAATTCCATGG CACCGT-3′,
reverse 5′-TGGACTCCACGACGTACTCA-3′;
Galectin-1 forward 5′-GGAGGCTGTCTTTCCCTTCC-3′,
reverse 5′-GTTGATGGCCTCCAGGTTGA-3′;
Galectin-2 forward 5′-GCGAATCCACCATTGTCTGC-3′,
reverse 5′-GGCTGAAGCACAGGTGATCT-3′;
Galectin-3 forward 5′-GAGCACCTGGAGCTTATCCC-3′,
reverse 5′-GCACTTGGCTGTCCAGAAGA-3′;
Galectin-8 forward 5′-CGGTAATCCCGTTTGTTGGC-3′,
reverse 5′-CACCTGGAATCTGTCTGCGT-3′;
Galectin-9 forward 5′-GCCCCTGGACAGATGTTCTC-3′,
reverse 5′-CAGTGCCTGACAGGAGGATG-3′;
Galectin-10 forward 5′-TGACAATCAAAGGGCGAC CA-3′,
reverse 5′-CGACGACCAAAGCACACTTG-3′;
Galectin-13 forward 5′-TGTCTTCTTTACCCGTGC CA-3′,
reverse 5′-TGCAGCTGTGGGTCATTGAT-3′

### Gelatin Zymography

Cells were cultured in serum-free medium for 24 h. The supernatant was collected after being centrifuged at 3,000 *g* for 15 min to remove the cell fragment. Then, the supernatant was concentrated by 10 times using Amicon Utra-0.5 10 kDa Centrifugal Filter Unit (Merck Millipore, Darmstadt, Germany). The protein concentration was measured by BSA kit (MultiSciences Biotech Co., Hangzhou, China). Here, 15 μg protein was separated by 7.5% SDS-polyacrylamide gel containing gelatin. Then, the gel was washed with washing buffer [50 mmol/L (mM) Tris-HCl, pH 7.5, containing 2.5% Triton X-100, 5 mM CaCl_2_, 1 μmol/L (μM) (ZnCl_2_)] at room temperature and incubated in incubating buffer (50 mM Tris-HCl, pH 7.5, containing 1% Triton X-100, 5 mM CaCl_2_, 1 μM ZnCl_2_) at 37°C for 24 h. After that, the gel was dyed by Coomassie blue staining solution (Solarbio, Beijing, China) for 1 h and was decolored by destaining solution (containing 40% methanol, 10% acetic acid) until white bands were visible.

### Transwell Migration and Invasion Assays

Cells were harvested and seeded in the upper chamber with serum-free medium. For invasion assay, the upper chamber was precoated with Matrigel (BD, Franklin Lakes, United States). And the lower chamber contained 500 μl complete medium. After 8 h for migrating or 24 h for invading, the bottoms of the inserts were fixed and stained in a solution containing 0.1% Gentian violet and 20% methanol. Cells were photographed (50×, 100×) and counted (100×) with an inverted phase contrast microscope (Leica, Wetzlar, Germany).

### Statistical Analysis

Kolmogorov–Smirnov test was used to test the normal distribution of the data. Levene test was used to test the homogeneity of variance. Student *t*-test and Mann–Whitney test were used for data comparison. *p* < 0.05 was considered significant. SPSS (International Business Machines Co., Armonk, United States) was used for statistical analysis.

## Results

### Placental Galectin-14 Is Dysregulated in Early Pregnancy Loss and Preeclampsia

Placenta tissues were obtained from women with early pregnancy loss, preeclampsia, and their controls. Demographic characteristics of the subjects were described in Tables 1, 2. There were no significant differences in maternal age and the gestational age at curettage between early pregnancy loss and its control. There were no significant differences in maternal age and fetal gender between severe preeclampsia and its control. The significant differences in blood pressure and proteinuria were consistent with the diagnosis of preeclampsia. The differences in gestational age at delivery and neonatal birth weight were due to preeclampsia. The difference in body mass index (BMI) was in accordance with the conclusion obtained in previous research that higher BMI is a risk factor for preeclampsia (Bartsch et al., 2016) (Table 2).

**FIGURE 1:**
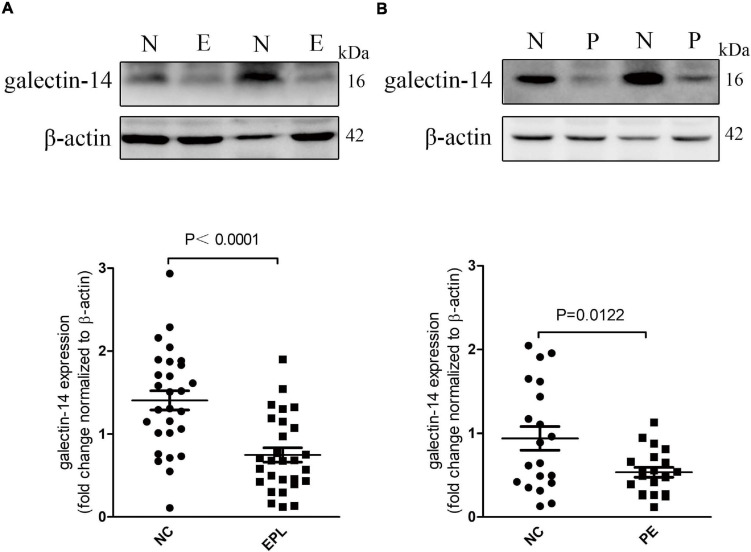
Galectin-14 is decreased in early pregnancy loss villi and preeclampsia placenta. **(A,B)** The expression level changes of galectin-14 in early pregnancy loss villi (*n* = 28) **(A)** and preeclampsia placentae (*n* = 20) **(B)** were determined by Western blotting. Dots in the graphs represent the number of biological samples analyzed. Results are presented as mean ± SEM. Statistical analysis: Student’s *t*-test. N or NC, control group; EPL or E, early pregnancy loss villi; P, preeclampsia placentae.

Galectin-14 expression was determined by Western blotting. Placental expression of galectin-14 was significantly lower in both early pregnancy loss and preeclampsia as compared with their controls (Figure 1). The dysregulation of placental galectin-14 in early pregnancy loss and preeclampsia implies its possible role in the pathogenesis of pregnancy complications and in the regulation of trophoblasts.

### Galectin-14 Promotes Trophoblast Migration and Invasion

**FIGURE 2:**
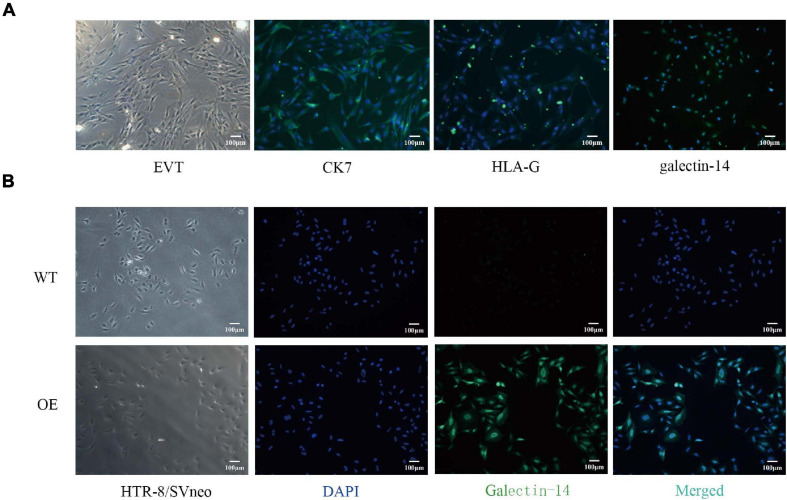
Distribution of galectin-14 in primary extravillous trophoblasts (EVTs) and galectin-14-overexpressed HTR-8/SVneo cell. **(A)** The primary EVTs were immunofluorescent stained by CK-7, HLA-G, and galectin-14 antibodies. **(B)** HTR-8/SVneo cell was transfected with flag-tagged galectin-14 vector or with empty vehicle. The location of galectin-14 in HTR-8/SVneo cell was identified by immunofluorescent method. Scale bar: 100 μm. EVT, primary extravillous trophoblast; CK7, cytokeratin-7; HLA-G, human leukocyte antigen-G; WT, HTR-8/SVneo transfected with empty vehicle; OE, galectin-14-overexpressed HTR-8/SVneo.

**FIGURE 3:**
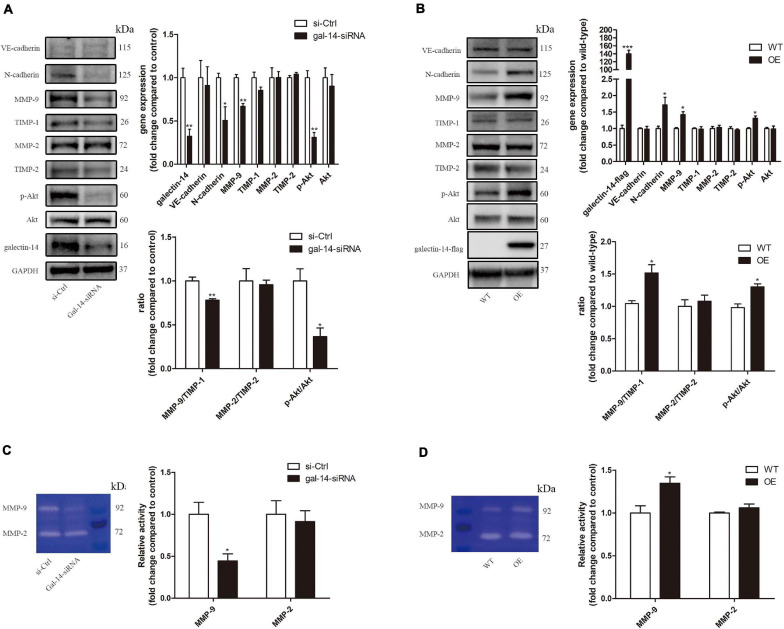
Galectin-14 promotes the expression of N-cadherin and matrix metalloproteinase (MMP)-9, Akt phosphorylation, and activity of MMP-9 in trophoblasts. **(A,C)** Primary extravillous trophoblasts (EVTs) were transfected with scramble siRNA or siRNA targeting galectin-14. **(B,D)** HTR-8/Svneo was transfected with empty vehicle or vector containing galectin-14 gene. **(A,B)** Protein expression was assessed by Western blotting with antibodies against VE-cadherin, N-cadherin, MMP-9, tissue inhibitor matrix metalloproteinase (TIMP)-1, MMP-2, TIMP-2, p-Akt, Akt, and galectin-14 and normalized to glyceraldehyde 3-phosphate dehydrogenase (GAPDH). **(C,D)** Gelatinase activity was determined by gelatin zymography. Results are presented as mean ± SEM of biological triplicates. Statistical analysis: Student’s *t*-test. *p < 0.05, **p < 0.01, ***p < 0.001. si-Ctrl, cells transfected with scramble siRNA; gal-14-siRNA, cells transfected with siRNA targeting galectin-14; WT, HTR-8/SVneo transfected with empty vehicle; OE, galectin-14-overexpressed HTR-8/SVneo.

**FIGURE 4:**
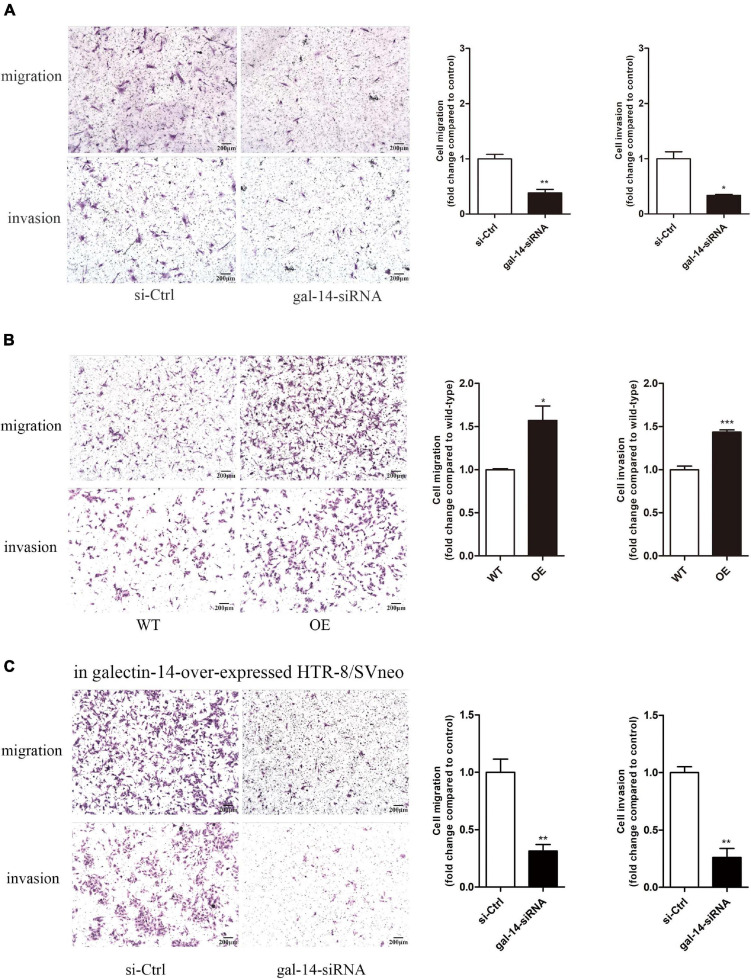
Galectin-14 promotes trophoblast migration and invasion. **(A)** Primary extravillous trophoblasts (EVTs) were transfected with scramble siRNA or siRNA targeting galectin-14. **(B)** HTR-8/Svneo was transfected with empty vehicle or vector containing galectin-14 gene. **(C)** The galectin-14-overexpressed HTR/SVneo was transfected with scramble siRNA or siRNA targeting galectin-14. **(A–C)** The migration and invasion ability of EVTs were examined using transwell migration and invasion assays. Results are represented as mean ± SEM of biological triplicates. Statistical analysis: Student’s *t*-test. *p < 0.05, **p < 0.01, ***p < 0.001. Scale bar: 200 μm. si-Ctrl, cells transfected with scramble siRNA; gal-14-siRNA, cells transfected with siRNA targeting galectin-14; WT, HTR-8/SVneo transfected with empty vehicle; OE, galectin-14-overexpressed HTR-8/SVneo.

With RT-PCR, the expression of galectin-14 was detected in primary EVTs but not in trophoblast cell lines (Supplementary Figure S). These results were confirmed in primary EVTs and HTR-8/SVneo by immunofluorescence (Figures 2A,B) and by Western blotting (Figure 3). siRNA-mediated galectin-14 knockdown significantly decreased the migration and invasion of primary EVTs (Figure 4A). The overexpression of galectin-14 by using lentivirus transfection significantly enhanced the migration and invasion of HTR-8/SVneo cells (Figure 4B). When the overexpression of galectin-14 was knocked down by siRNA, the enhancement of migration and invasion ability was abrogated (Figure 4C).

### Galectin-14 Promotes the Expression of N-Cadherin and Matrix Metalloproteinase 9 in Trophoblasts

The expressions of N-cadherin, VE-cadherin, E-cadherin, MMP-2, MMP-9, TIMP-2, and TIMP-1 were examined in galectin-14 knockdown primary EVTs and galectin-14-overexpressed HTR-8/SVneo. A decrease in N-cadherin, MMP-9 expression, MMP-9 activity, and MMP-9/TIMP-1 ratio was observed in galectin-14-knockdown primary EVTs (Figures 3A,C). On the contrary, an upregulation of N-cadherin, MMP-9 expression, MMP-9 activity, and MMP-9/TIMP-1 ratio was detected in galectin-14-overexpressed HTR-8/SVneo (Figures 3B,D). No significant change of VE-cadherin expression was detected. E-cadherin was not detectable in HTR-8/SVneo and in human primary EVTs, which might due to the hypermethylation of the promoter (Chen et al., 2013; Cheng et al., 2013; Szklanna et al., 2017).

### N-Cadherin and Matrix Metalloproteinase 9 Contribute to the Promotion of Trophoblast Migration and Invasion

**FIGURE 5:**
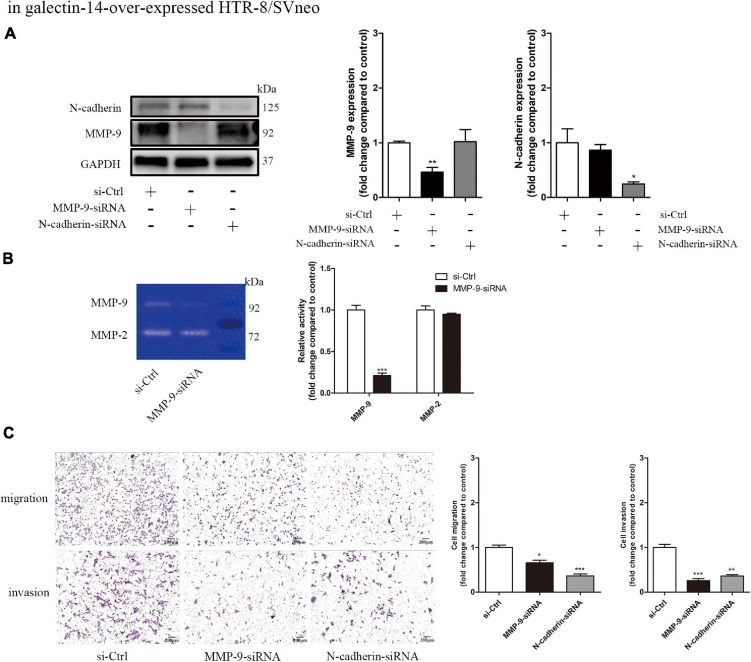
Knockdown of matrix metalloproteinase (MMP)-9 and N-cadherin decreases the migration and invasion of galectin-14-overexpressed HTR/Svneo. siRNA-mediated MMP-9 and N-cadhrin knockdown was performed in galectin-14-overexpressed HTR-8/Svneo. **(A)** Protein expression was assessed by Western blotting with antibodies against N-cadherin and MMP-9 and normalized to glyceraldehyde 3-phosphate dehydrogenase (GAPDH). **(B)** Gelatinase activity was determined by gelatin zymography. **(C)** The migration and invasion ability of extravillous trophoblasts (EVTs) were examined using transwell migration and invasion assays. Results are represented as mean ± SEM of biological triplicates. Statistical analysis: Student’s *t*-test. *p < 0.05, **p < 0.01, ***p < 0.001. Scale bar: 200 μm. si-Ctrl, cells transfected with scramble siRNA; MMP-09-siRNA, cells transfected with siRNA targeting MMP-9; N-cadherin-siRNA, cells transfected with siRNA targeting N-cadherin.

To investigate the involvement of N-cadherin and MMP-9 upregulation in the promotion of trophoblast migration and invasion, we performed siRNA-mediated knockdown of N-cadherin and MMP-9 in galectin-14-overexpressed HTR-8/SVneo. The N-cadherin and MMP-9 expression reduced by 75 and 60%, respectively (Figure 5A). The activity of MMP-9 reduced by 70–90% (Figure 5B). Knockdown of MMP-9 and N-cadherin decreased migration and invasion of HTR-8/SVneo (Figure 5C).

### Akt Signaling Is Involved in Galectin-14 Promotion of Matrix Metalloproteinase 9 Expression

**FIGURE 6:**
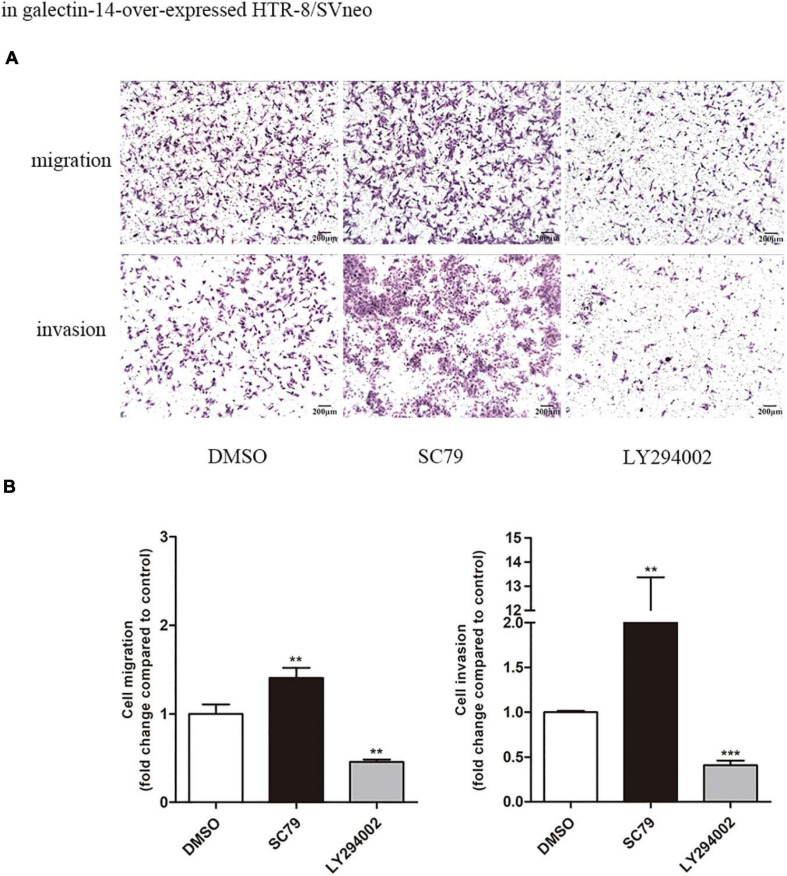
Increased phosphorylation of Akt promotes trophoblast migration and invasion in HTR-8/SVneo. In galectin-14-overexpressed HTR-8/SVneo, 10 μM SC79 or 10 μM LY294002 was added as the agonist or inhibitor of Akt phosphorylation. **(A)** The migration and invasion ability of extravillous trophoblasts (EVTs) were examined using transwell migration and invasion assays. **(B)** Results are represented as mean ± SEM of biological triplicates. Statistical analysis: Student’s *t*-test. **p < 0.01, ***p < 0.001. Scale bar: 200 μm. Dimethylsulfoxide (DMSO), cells added with DMSO of the same volume; SC79, cells added with 10 μM SC79; LY294002, cells added with 10 μM LY294002.

**FIGURE 7:**
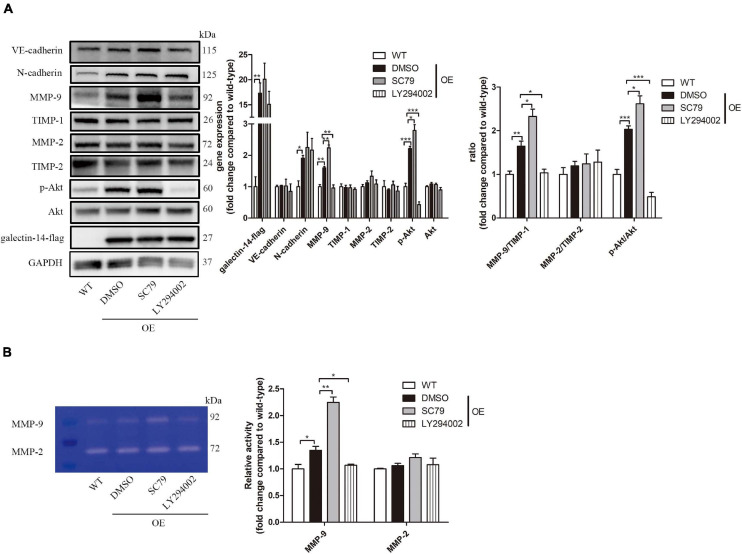
Galectin-14 promotes matrix metalloproteinase (MMP)-9 expression and activity through enhancing Akt phosphorylation. In galectin-14-overexpressed HTR-8/SVneo, 10 μM SC79 or 10 μM LY294002 was added as the agonist or inhibitor of Akt phosphorylation. **(A)** Protein expression was assessed by Western blotting with antibodies against VE-cadherin, N-cadherin, MMP-9, tissue inhibitor matrix metalloproteinase (TIMP)-1, MMP-2, TIMP-2, p-Akt, Akt, and galectin-14 and normalized to glyceraldehyde 3-phosphate dehydrogenase (GAPDH). **(B)** Gelatinase activity was determined by gelatin zymography. Results are presented as mean ± SEM of biological triplicates. Statistical analysis: Student’s *t*-test. *p < 0.05, **p < 0.01, ***p < 0.001. WT, HTR-8/SVneo transfected with empty vehicle; OE, galectin-14-overexpressed HTR-8/SVneo. Dimethylsulfoxide (DMSO), cells added with DMSO of the same volume; SC79, cells added with 10 μM SC79; LY294002, cells added with 10 μM LY294002.

Inhibition of Akt phosphorylation at Ser473 was observed in galectin-14-knockdown primary EVTs, while activation was detected in galectin-14-overexpressed HTR-8/SVneo (Figures 3A,B). Akt phosphorylation agonist (SC79) enhanced the cell migration and invasion ability (Figure 6) and increased MMP-9 expression and activity and MMP-9/TIMP-1 ratio (Figure 7). On the contrary, Akt inhibitor (LY294002) inhibited the cell migration and invasion (Figure 6) and decreased MMP-9 expression and activity and MMP-9/TIMP-1 ratio (Figure 7). No significant change was detected in N-cadherin expression. These findings imply that enhancement of Akt phosphorylation is necessary for the promotion of MMP-9 by galectin-14.

## Discussion

The dysfunction of trophoblasts is one of the major pathogeneses of early pregnancy loss and preeclampsia. In the current investigation, we found the decreased placental expression pattern of galectin-14 in early pregnancy loss and preeclampsia, which were consistent with previous studies (Than et al., 2014; Fritz et al., 2015; Balogh et al., 2019). These findings point to the importance of galectin-14 in these pregnancy-related diseases. We further demonstrated that galectin-14 promoted the migration and invasion of trophoblasts through enhancing the expression of MMP-9 and N-cadherin.

In the placenta, galectin-14 is expressed in STB and EVT and is necessary for the maintenance of immune tolerance at the fetal–maternal interface (Than et al., 2009, 2014; Balogh et al., 2019). However, little is known about the regulatory effects of galectin-14 on trophoblasts. In this study, the effects of galectin-14 knockdown or overexpression on trophoblast migration and invasion indicate the positive impact of galectin-14 on trophoblast migration and invasion. Successful trophoblast migration and invasion play a key role in embryonic implantation and establishment of healthy pregnancy. Thus, the dysregulation of galectin-14 in trophoblasts is involved in the pathogenesis of early pregnancy loss and preeclampsia. In addition, unlike the recombinant galectin-14 used in the previous researches, our results came from the direct knockdown of endogenic galectin-14 in primary EVTs, which are more convincing. And we further established a galectin-14-synthesizing trophoblast cell line for studies on mechanisms.

Gelatinases, including MMP-2 and MMP-9, degrade extracellular matrix (ECM) to facilitate trophoblastic migration and invasion (Shimonovitz et al., 1994). The activity of MMP-2 and MMP-9 is inhibited by TIMP-2 and TIMP-1. The different temporal expression patterns during pregnancy indicate that MMP-2 is mainly involved in embryonic implantation while MMP-9 in trophoblast invasion (Espino et al., 2017). In the placenta, MMP-9 is expressed in the EVT-rich regions (Naruse et al., 2009). *In vivo*, MMP-9-null mouse embryos showed deficiency of trophoblast invasion (Plaks et al., 2013). The decrease in MMP-2 and MMP-9 activity or ratios of MMP-2/TIMP-2 and MMP-9/TIMP-1 might be involved in the pathogeneses of early pregnancy loss and preeclampsia (Graham and McCrae, 1996; Choi et al., 2003; Shokry et al., 2009; Plaks et al., 2013; Gutierrez et al., 2020; Lin et al., 2020). Hereby, we found that galectin-14 promoted the expression and activity of MMP-9 and increased the MMP-9/TIMP-1 ratio while the migration and invasion of trophoblasts were enhanced.

Epithelial–mesenchymal transition (EMT) is another indispensable process for trophoblasts obtaining motility and invasion capacity. During EMT, trophoblasts lose their endothelial phenotype, loosen intercellular adhesion, and modulate cytoskeleton. The cell–cell junction proteins, such as E-cadherin, are degraded, while mesenchymal cell markers, such as N-cadherin and VE-cadherin, are upregulated (Kokkinos et al., 2010). The temporal and spatial expression of cadherins is the hallmark of trophoblast phenotype transformation, and defects in this process result in shallow trophoblast invasion, which are associated with pregnancy complications, such as early pregnancy loss and preeclampsia (Brown et al., 2005; Multhaup et al., 2018). In mice, galectin-1 improves trophoblast invasion and migration by increasing N-cadherin expression and decreasing E-cadherin expression (You et al., 2018). Herein, we observed that galectin-14 enhanced the expression of N-cadherin; meanwhile, it prompted the invasion and migration of trophoblasts, indicating that, like galectin-1, galectin-14 facilitates migration and invasion by promoting EMT in trophoblasts.

Our results indicate that galectin-14 promotes trophoblast migration and invasion through enhancing EMT and increasing the expression and activity of MMP-9. Proteomics analysis of galectin-14-overexpressed trophoblast cell line revealed the involvement of PI3K–Akt signaling pathway (data not shown). There is a significantly decreased expression of p-Akt in the villi of early pregnancy loss (Zhang et al., 2017). In our study, we found that galectin-14 promoted the phosphorylation of Akt. Increasing the phosphorylation of Akt promoted trophoblast migration and invasion and MMP-9 expression and activity, but not N-cadherin. These data suggest that galectin-14 enhances MMP-9 expression and activity through promoting Akt phosphorylation, while the mechanism through which galectin-14 promotes N-cadherin expression remains to be studied.

An *in vivo* research will enhance our insight into the roles of galectin-14 in pregnancy, placental development, and pathogeneses of early pregnancy loss and preeclampsia. However, there is no homologous gene of *LGALS14* (the gene of galectin-14) in rat or mouse, which hammered the investigation with animal models.

**FIGURE 8:**
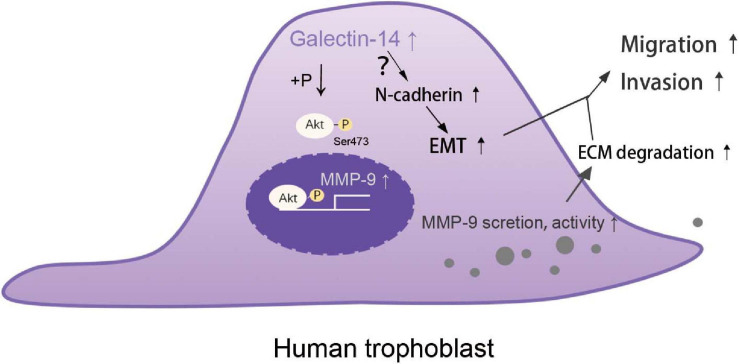
Schematic representation of galectin-14 enhancing trophoblast migration and invasion. The expression of galectin-14 during trophoblast differentiation promotes Akt phosphorylation, leading to increasing matrix metalloproteinase (MMP)-9 expression, secretion, and activity, thus increasing the degradation of extracellular matrix (ECM). Galectin-14 also promotes epithelial–mesenchymal transition (EMT) by upregulating N-cadherin expression. Together, the increased ECM degradation and EMT help trophoblasts obtain the capacity of migration and invasion.

In summary, galectin-14 promotes trophoblast migration and invasion by enhancing the expression of MMP-9 and N-cadherin through Akt phosphorylation (Figure 8). The dysregulation of galectin-14 is involved in the pathogenesis of early pregnancy loss and preeclampsia.

## Data Availability Statement

The original contributions presented in the study are included in the article/Supplementary Material, further inquiries can be directed to the corresponding author/s.

## Ethics Statement

The studies involving human participants were reviewed and approved by Institutional Ethical Committee of Women’s hospital, School of Medicine, Zhejiang University. The patients/participants provided their written informed consent to participate in this study.

## Author Contributions

MW, LB, and MD conceived and designed the experiments. MW, PW, YaX, and PJ performed the experiments and analyzed the data. YuX and ZW collected the samples and consents. MW wrote the draft. LB, YQ, and MD edited the manuscript. All authors contributed to the article and approved the submitted version.

## Conflict of Interest

The authors declare that the research was conducted in the absence of any commercial or financial relationships that could be construed as a potential conflict of interest.
