# Supplementary material for: Galectin-14 Promotes Trophoblast Migration and Invasion by Upregulating the Expression of MMP-9 and N-Cadherin
Source: Front Cell Dev Biol. 2021 Mar 16;9:645658. doi: 10.3389/fcell.2021.645658 (PMC8007908; doi:10.3389/fcell.2021.645658)
Supplement: Supplementary file 1 [file Presentation_1.PPTX]

## Slide 1
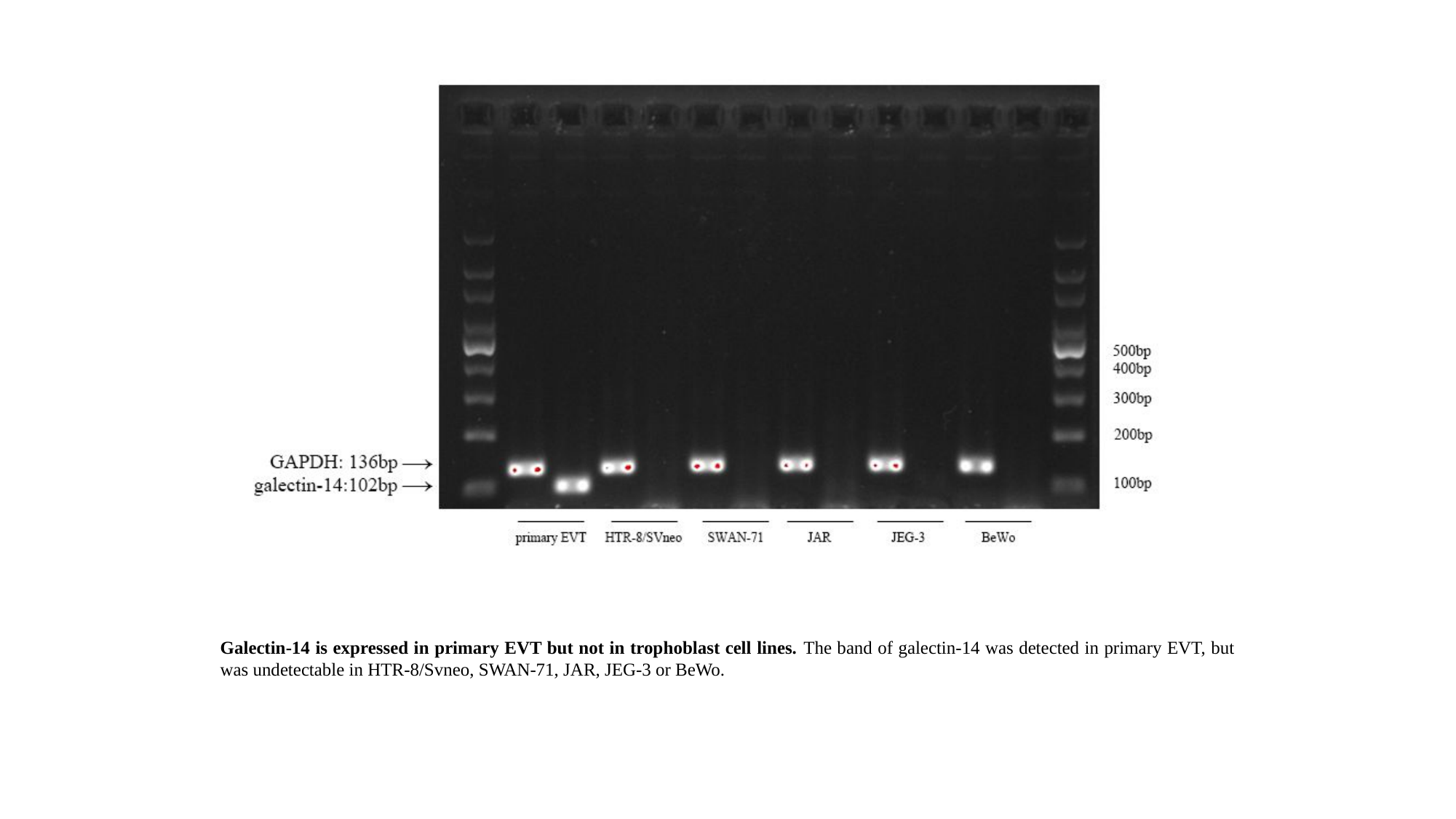

Galectin-14 is expressed in primary EVT but not in trophoblast cell lines. The band of galectin-14 was detected in primary EVT, but was undetectable in HTR-8/Svneo, SWAN-71, JAR, JEG-3 or BeWo.

## Slide 2
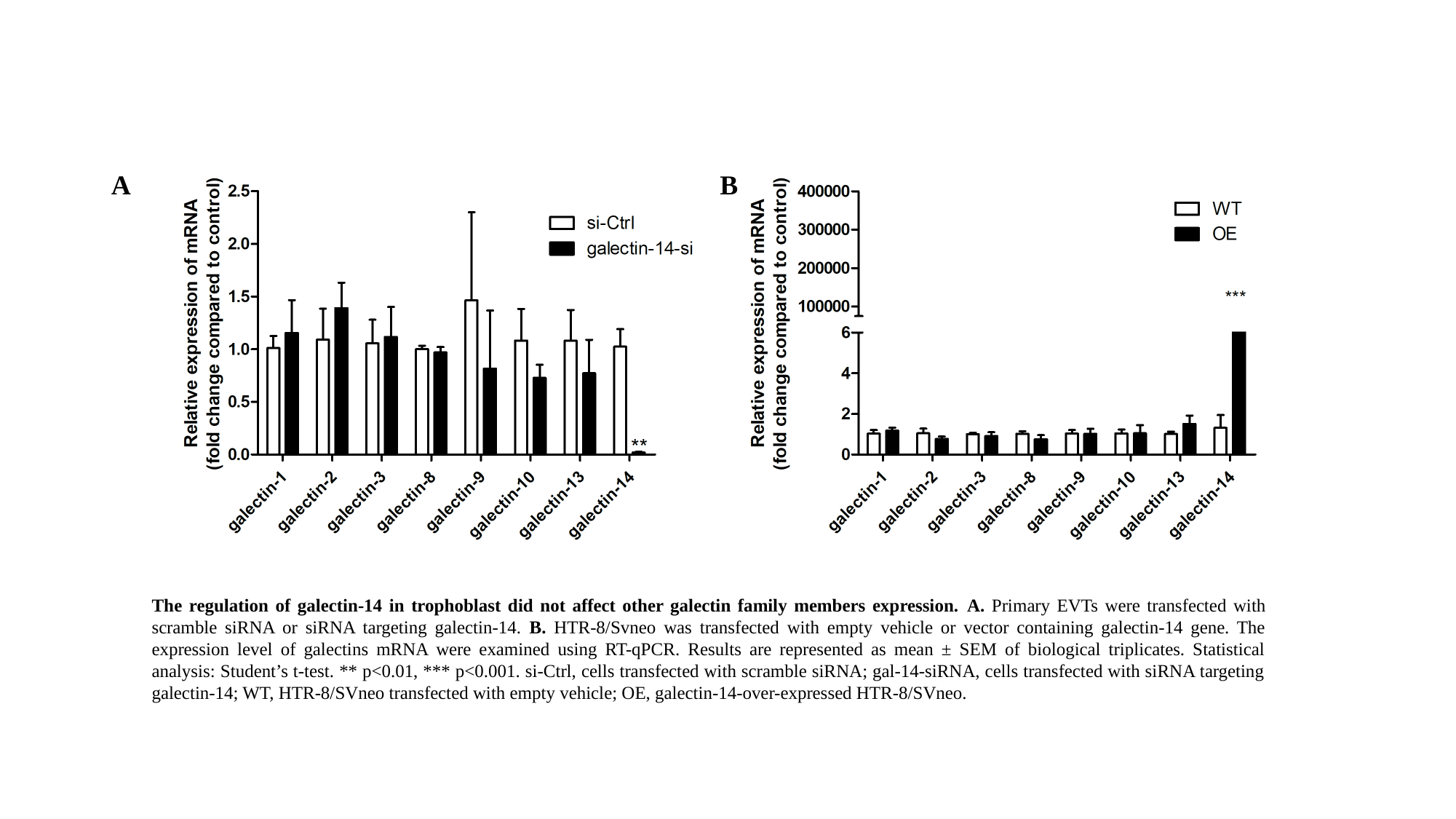

A
B
The regulation of galectin-14 in trophoblast did not affect other galectin family members expression. A. Primary EVTs were transfected with scramble siRNA or siRNA targeting galectin-14. B. HTR-8/Svneo was transfected with empty vehicle or vector containing galectin-14 gene. The expression level of galectins mRNA were examined using RT-qPCR. Results are represented as mean ± SEM of biological triplicates. Statistical analysis: Student’s t-test. ** p<0.01, *** p<0.001. si-Ctrl, cells transfected with scramble siRNA; gal-14-siRNA, cells transfected with siRNA targeting galectin-14; WT, HTR-8/SVneo transfected with empty vehicle; OE, galectin-14-over-expressed HTR-8/SVneo.
